# The association of hearing loss with frailty among community-dwelling older adults: findings from the National Health and Aging Trends Study

**DOI:** 10.1186/s12877-023-04465-1

**Published:** 2023-11-17

**Authors:** Sahar Assi, Wuyang Zhang, John P. Carey, Jennifer A. Deal, Alison Huang, Esther S. Oh, Pablo Martinez-Amezcua, Nicholas S. Reed

**Affiliations:** 1 Cochlear Center for Hearing and Public Health, Johns Hopkins University, 2024 E Monument St, Baltimore, MD 21205 USA; 2 Department of Epidemiology, Johns Hopkins Bloomberg School of Public Health, Baltimore, MD USA; 3 Department of Otolaryngology-Head and Neck Surgery, Johns Hopkins University School of Medicine, Baltimore, MD USA; 4 Division of Geriatric Medicine and Gerontology, Johns Hopkins University School of Medicine, Baltimore, MD USA

**Keywords:** Frailty, Hearing loss, Hearing aids

## Abstract

**Background:**

The identification of modifiable risk factors is crucial for the prevention and/or reversal of frailty, which is associated with significant morbidity and mortality. Hearing loss affects two-thirds of older adults in the United States (U.S.) and is associated with physical and cognitive decline which may increase frailty risk. We investigated the association of hearing loss and hearing aid use with frailty and pre-frailty in a nationally representative sample of older adults in the U.S.

**Methods:**

Cross-sectional analysis of the National Health and Aging Trends Study (2021 round). The better-hearing ear pure-tone average (BPTA) at speech-frequencies (0.5–4 kHz) was modeled continuously (per 10 dB) and categorically (no ≤ 25 dB, mild 26–40 dB, moderate or greater > 40 dB hearing loss). Hearing aid use was self-reported. The physical frailty phenotype (frail, pre-frail, robust) was determined based on Fried criteria: unintentional weight loss, exhaustion, low physical activity, weakness, slow walking speed. We used multinomial multivariable regression adjusted for sociodemographic and health characteristics (odds ratios [95% Confidence Intervals]).

**Results:**

Among 2,361 participants (mean age = 81 years, 56% female, 19% Black), 860 (36%) had mild and 864 (37%) had moderate or greater hearing loss. Worse hearing was associated with greater odds of being frail versus robust (OR = 1.20 [1.05–1.38] per 10 dB difference). Categorically, moderate or greater hearing loss was associated with greater odds of being frail (OR = 1.84 [1.01–3.08]) and pre-frail (OR = 1.46 [1.01–2.10]) versus robust. Among 1,724 participants with hearing loss, compared to hearing aid users (*N* = 522), nonusers had greater odds of being frail (OR = 2.54 [1.54–4.18]) and pre-frail (OR = 1.51 [1.05–2.17]) versus robust, and frail versus pre-frail (OR = 1.68 [1.04–2.72]).

**Conclusions:**

In a nationally representative sample of older adults in the U.S., using gold-standard hearing measures and a validated frailty phenotype, hearing loss and lack of hearing aid use was cross-sectionally associated with frailty and pre-frailty. Future longitudinal studies are needed to establish if hearing loss is a risk factor for frailty, which may have significant clinical importance.

**Supplementary Information:**

The online version contains supplementary material available at 10.1186/s12877-023-04465-1.

## Introduction

Frailty is a syndrome characterized by diminished physiological reserve causing increased vulnerability to adverse events following stressors and occurs in nearly 1 in 6 older adults in the United States (U.S.) [1]. Frail individuals are at higher risk for falls, hospitalization, and death [2–5]. Importantly, frailty may be reversed or attenuated by interventions, including nutrition support, reduction in polypharmacy, exercise, and vitamin D supplementation [6]. Further identification of modifiable risk factors for frailty among older adults is of significant clinical and public health importance.

Almost two-thirds of adults older than 70 years in the U.S. have hearing loss [7]. It is independently associated with falls [8], poorer physical function [9, 10], social isolation [11], and cognitive decline [12]. Hearing loss could also be associated with frailty through the aforementioned outcomes; however, there is a paucity of evidence. A recent systematic review identified only 13 studies on the topic [13], the majority of which were in non-US representative population-based and clinical samples that may limit generalizability of findings or used self-report hearing measures that are not sensitive as clinical measures [14]. Furthermore, hearing interventions facilitate communication and cognitive processing among older adults with hearing loss, which may attenuate the associations with hearing loss-related outcomes such as cognitive decline [15]. Yet, few studies have looked at whether hearing aid use modifies the association of hearing loss with frailty.

The National Health and Aging Trends Study (NHATS), a nationally representative study of older adults in the United States, added clinical gold standard audiometric measures in Round 11 (2021) to a test battery that already included a well-characterized frailty measure. Using this data, we tested the null hypothesis that the odds of being frail and pre-frail versus robust and frail versus pre-frail are similar among older adults with and without hearing loss. In secondary analyses, we tested the null hypothesis that among older adults with hearing loss, hearing aid users and nonusers have similar odds of being frail and pre-frail versus robust and frail versus pre-frail.

## Methods

### Study population

The National Health and Aging Trends Study (NHATS) is a nationally representative cohort study of Medicare beneficiaries ages 65 and older in contiguous U.S. since 2011 [16]. Importantly, the NHATS study design oversamples by age (90 years and older) and race (Black individuals) and uses in-home visits that overcome mobility and travel restrictions. Data are collected through annual in-person interviews. In 2021 (Round 11), 2803 participants had audiometric hearing assessments. We excluded institutionalized participants (*n* = 183) and participants missing data on sociodemographic (*n* = 28 race, *n* = 2 education) or health characteristics (*n* = 1 hypertension, *n* = 2 stroke, *n* = 27 BMI), and frailty status (*n* = 50) resulting in a sample of 2,510 participants. We additionally excluded 149 participants with insufficient frailty data to distinguish whether they were pre-frail or frail. Our final analytic sample consisted of 2,361 participants. The NHATS was approved by the Institutional Review Board at the Johns Hopkins Bloomberg School of Public Health. All participants provided informed consent at the time of enrollment in the study. The data used are de-identified and publicly available.

### Assessment of frailty

We used the physical frailty phenotype from the Cardiovascular Health Study [17] to assess frailty. The phenotype has five components: unintentional weight loss, exhaustion, low physical activity, weakness, and slow walking speed. The operationalization of each frailty component that we used has been previously used in NHATS [1]. “Unintentional weight loss” was defined as having a body mass index < 18.5 kg/m^2^ or unintentionally losing ≥ 10 pounds in the last year. “Exhaustion” was present when participants reported low energy or limited activities due to being easily exhausted. “Low physical activity” was present when participants reported not engaging in vigorous activities or walking for exercise in the last month. “Weakness” was defined by having a grip strength lower than the weight-and-sex specific bottom quintile in the weighted population distribution, based on the maximum over two trials using the dominant hand. “Slow walking speed” was defined as having a gait speed slower than the bottom height-and-sex specific quintile, based on the first of two usual-pace walking trials. The two latter criteria were derived based on the distribution of participants at Round 11 and are detailed in Supplementary Tables (Table S1 and Table S2). For the grip strength and gait speed assessments, participants who were not tested due to safety concerns, were ineligible because of recent surgery or pain, or were unable to complete the test were given a score of “0”, following NHATS recommendations [1, 18]. Participants were categorized as frail if they met criteria for 3 or more components, pre-frail if 1–2 components, and robust if they didn’t meet criteria for any of the components.

### Assessment of hearing

Pure-tone air-conduction audiometry was done using iPad-based portable audiometers (SHOEBOX Ltd, Ontario, Canada) with sound attenuating Sennheiser DD450 headphones in participants’ homes by trained technicians [19]. The application was validated against gold-standard sound booth audiometry [19–21]. The lowest volume (in decibels hearing level [dB HL]) at which participants responded to the presented tones in each ear separately, without hearing aids (if applicable), was identified. A pure-tone average (PTA) of the frequencies most important for speech understanding (0.5, 1, 2, and 4 kHz) was derived for each ear, consistent with previous population-level definitions of hearing loss. We used the better-hearing ear PTA (BPTA, i.e., the ear with the lower PTA) continuously, scaled by 10 dB HL, and categorically using the clinical cut points most commonly used in population-level research as no hearing loss: ≤ 25 dB HL, mild hearing loss 26–40 dB HL, and moderate or greater hearing loss: > 40 dB HL (the latter group included those with severe and profound hearing loss because the sample size was too small for separate categories) [22]. In sensitivity analyses, we categorized the PTA using the recently adopted World Health Organization categories (no hearing loss: < 20 dB HL, mild hearing loss: 20–34.9 dB HL, moderate hearing loss: 35–49.9 dB HL, moderately severe hearing loss: 50–64.9 dB HL, and severe or greater hearing loss: > 64.9 dB HL). Information on use of hearing devices was collected by asking “In the last month, have you used a hearing aid or other hearing device?” (yes/no).

### Covariates

Sociodemographic characteristics included age (years), sex (male/female), race/ethnicity (White, Black, Other), educational attainment (less than high school, high school, some college, college or more), and total income (incomplete measures were replaced by values imputed by NHATS [23]). Health characteristics included BMI, calculated based on self-reported height and weight, and self-reported physician diagnoses of hypertension, diabetes, and stroke. Specifically, participants were asked “Since the last interview, has a doctor told you that you had hypertension/diabetes/stroke? (yes/no/previously reported)”. Participants were categorized as having the condition if they answered positively in the current or any of the previous rounds of data collection.

### Statistical analysis

We summarized participants’ characteristics across hearing loss categories using means and standard deviations for continuous variables and frequencies and percentages for categorical ones. We compared characteristics across hearing loss categories using one-way ANOVA for continuous variables and chi-squared tests for categorical ones. We used multinomial logistic regression models to examine the association between hearing loss and frailty. We estimated the odds of being frail and pre-frail versus robust (reference) and frail versus pre-frail (reference) for each 10 dB HL increase in BPTA and across hearing loss categories. We adjusted for sociodemographic (age, sex, race/ethnicity, educational attainment, and natural log transformed income) and health (BMI, hypertension, diabetes, and stroke) characteristics. In secondary analyses, we first explored the association of hearing loss and frailty stratified by two age groups (71–80 and > 80 years) to account for residual confounding by age. Second, in samples restricted to participants with mild or greater hearing loss (BPTA > 25 dB HL, *N* = 1,724) and moderate or greater hearing loss (BPTA > 40 dB HL, *N* = 864), we used the same models to estimate the odds of being frail and pre-frail versus robust (reference) and frail versus pre-frail (reference) by hearing aid use and additionally adjusted for BPTA. We present results as odds ratios (OR) with 95% Confidence Intervals (CI). To obtain representative estimates of US older adults, we accounted for sampling weights [16]. All analyses were conducted using Stata version 17.0 (StataCorp, TX).

### Sensitivity analyses

To explore whether including participants with insufficient frailty data (*n* = 149) in our analytic sample changed our estimates, we first considered them pre-frail and estimated the relative odds of being frail and pre-frail versus robust and frail versus pre-frail across hearing measures (*N* = 2,510) and by hearing aid use (the latter among participants with mild or greater hearing loss, *N* = 1,833) using the previously described models. Second, we considered them frail and repeated the same analyses.

## Results

### Sample characteristics

Among 2,361 participants (mean age = 81 years, 56% female, 19% Black), 637 (27%) did not have hearing loss, 860 (36%) had mild hearing loss, and 864 (37%) had moderate or greater hearing loss (Table 1). Among those with moderate or greater hearing loss, 131 had severe hearing loss and 22 had profound hearing loss. Participants with moderate or greater hearing loss were older (mean age = 84 years) and more often male (51%), White (79%), with lower education attainment, and more likely to have hypertension. Approximately 13% and 47% of those with mild and moderate or greater hearing loss, respectively, used hearing aids. About 18% (*n* = 424) were frail, 50% were pre-frail (*n* = 1,173), and 32% (*n* = 764) were robust. A greater proportion of those with hearing loss were frail and pre-frail. Specifically, 12% of those with no hearing loss were frail, while 17% of those with mild and 24% of those with moderate or greater hearing loss were frail. Similarly, 46% of those with no hearing loss were pre-frail compared to 50% and 53% of those with mild and moderate or greater hearing loss, respectively.

**Table 1:** Characteristics of community-dwelling older adults from the National Health and Aging Trends Study (2021) stratified by hearing status

	**Total**	**No hearing loss (BPTA ≤ 25 dB HL)**	**Mild hearing loss (BPTA 26–40 dB HL)**	**Moderate or greater hearing loss (BPTA > 40 dB HL)**	***p*-value**
**Number of observations, n (%)**	2,361 (100)	637 (27)	860 (36)	864 (37)	
**Age, mean (SD)**	81.1 (6.1)	77.9 (4.6)	80.3 (5.3)	84.1 (6.4)	< 0.001
**Female, n (%)**	1,314 (55.7)	403 (63.3)	492 (57.2)	419 (48.5)	< 0.001
**Race/ethnicity, n (%)**					< 0.001
White	1,762 (74.6)	451 (70.8)	630 (73.3)	681 (78.8)	
Black	446 (18.9)	150 (23.5)	178 (20.7)	118 (13.7)	
Other^a^	153 (6.5)	36 (5.7)	52 (6.0)	65 (7.5)	
**Educational attainment, n (%)**					< 0.001
Less than high school	340 (14.4)	62 (9.7)	126 (14.7)	152 (17.6)	
High school	599 (25.4)	136 (21.4)	214 (24.9)	249 (28.8)	
Some college	655 (27.7)	188 (29.5)	238 (27.7)	229 (26.5)	
College or more	767 (32.5)	251 (39.4)	282 (32.8)	234 (27.1)	
**Total reported income, mean (SD)**	68162.5 (148962.6)	75030.3 (84375.6)	73081.3 (222486.2)	58203.0 (77497.5)	0.046
**Hearing aid use, n (%)**	533 (22.6)	11 (1.7)	114 (13.3)	408 (47.2)	< 0.001
**Body Mass Index (kg/m**^**2**^**), mean (SD)**	27.5 (5.7)	27.7 (5.7)	27.9 (5.9)	27.1 (5.4)	0.007
**Hypertension, n (%)**	1,783 (75.5)	460 (72.2)	650 (75.6)	673 (77.9)	0.041
**Diabetes, n (%)**	691 (29.3)	173 (27.2)	271 (31.5)	247 (28.6)	0.16
**Stroke, n (%)**	45 (1.9)	12 (1.9)	19 (2.2)	14 (1.6)	0.67
**Frailty status, n (%)**					< 0.001
Robust	764 (32.4)	271 (42.5)	288 (33.5)	205 (23.7)	
Pre-frail	1,173 (49.7)	291 (45.7)	428 (49.8)	454 (52.5)	
Frail	424 (18.0)	75 (11.8)	144 (16.7)	205 (23.7)	

^a^Other: Hispanic and non-Hispanic American Indian, Asian, Native Hawaiian, and Pacific Islander

### Odds of being frail and pre-frail versus robust by hearing status

In the fully adjusted model, every 10 dB HL higher BPTA (i.e., worse hearing) was associated with a 20% increase in odds of being frail versus robust (OR = 1.20; 95% CI [1.05–1.38]) (Table 2). Estimates also suggest 10% increased odds of being pre-frail versus robust (OR = 1.10 [1.00–1.21] per 10 dB HL, *p*-value = 0.056) with worse hearing.

**Table 2:** Odds of being frail and pre-frail by hearing status among community-dwelling older adults from NHATS

	**Frail vs. Robust**		**Pre-frail vs. Robust**		**Frail vs. Pre-frail**
	**Odds Ratios (95% Confidence Intervals)**				
**Total sample (*N* = 2,361)**					
**BPTA**^**a**^**, per 10 dB HL**^**b**^	**1.20 (1.05–1.38)**	1.10 (1.00–1.21)		1.10 (0.97–1.25)	
**Age group 71–80 years (*N* = 1,211)**					
**BPTA**^**a**^**, per 10 dB HL**^**b**^	**1.31 (1.09–1.59)**		1.10 (0.97–1.24)		1.20 (0.99–1.45)
**Hearing loss categories**					
No hearing loss (*N* = 466)	Reference		Reference		Reference
Mild hearing loss (*N* = 476)	1.35 (0.76–2.37)		1.25 (0.88–1.78)		1.08 (0.62–1.87)
Moderate or greater hearing loss (*N* = 269)	**2.41 (1.20–4.82)**		1.42 (0.91–2.20)		1.70 (0.85–3.40)
**Age group > 80 years (*N* = 1,150)**					
**BPTA**^**a**^**, per 10 dB HL**^**b**^	1.05 (0.90–1.22)		1.06 (0.94–1.20)		0.99 (0.87–1.12)
**Hearing loss categories**					
No hearing loss (*N* = 171)	Reference		Reference		Reference
Mild hearing loss (*N* = 384)	1.08 (0.58–2.00)		1.37 (0.80–2.37)		0.79 (0.44–1.39)
Moderate or greater hearing loss (*N* = 595)	1.15 (0.64–2.07)		1.51 (0.92–2.48)		0.76 (0.45–1.29)

Adjusted for age, sex, race/ethnicity, educational attainment, log of income, BMI, hypertension, diabetes, and stroke

Reported as relative odds ratios from three-category multinomial model (robust, pre-frail, or frail)

*Abbreviations*: ^a^*BPTA* better-hearing ear pure-tone average, ^b^*dB HL* decibels hearing level

**Fig. 1:**
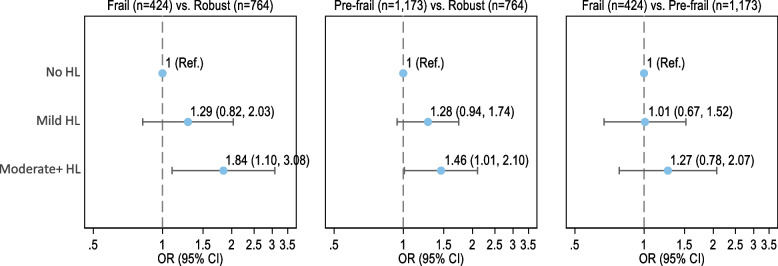
Multinomial model of frailty status by hearing loss categories among community-dwelling older adults from NHATS (*N* = 2,361). Legend: Estimates are odds ratios with no hearing loss group as the reference. Adjusted for sociodemographic (age, sex, race/ethnicity, educational attainment, and log 10 of income) and health (BMI, hypertension, diabetes, and stroke) characteristics. Abbreviations – CI: Confidence Intervals; HL: Hearing Loss; OR: Odds Ratios

Across hearing loss categories (Fig. 1), participants with moderate or greater hearing loss had increased odds of being frail (OR = 1.84 [1.10–3.08]) and pre-frail (OR = 1.46 [1.01–2.10]) versus robust compared to those without hearing loss. Although estimates for mild hearing loss were not statistically significant, findings suggest greater odds of being frail (OR = 1.29 [0.82–2.03]) and pre-frail (OR = 1.28 [0.94–1.74]) versus robust across hearing loss categories.

### Odds of being frail versus pre-frail by hearing status

Relative to being pre-frail, findings were suggestive of greater odds of being frail with worse hearing although not statistically significant (OR = 1.10 [0.97–1.25], per 10-dB HL) (Table 2). In addition, the odds of being frail versus pre-frail were not significantly greater across hearing loss categories (OR = 1.01 [0.67–1.52] for mild and 1.27 [0.78–2.07] for moderate or greater compared to no hearing loss) (Fig. 1).

### Odds of being frail and pre-frail stratified by age

Among older adults aged 71–80, every 10 dB HL higher BPTA was associated with a 31% increase in odds of being frail versus robust (OR = 1.31 [1.09–1.59], per 10-dB HL) (Table 2). Estimates were also suggestive of greater odds of being pre-frail versus robust (OR = 1.10 [0.97–1.24]) and frail versus pre-frail (OR = 1.20 [0.99–1.45]) but were not statistically significant. Across hearing loss categories, moderate or greater hearing loss was associated with greater odds of being frail versus robust (OR = 2.41 [1.20–4.82]). Among those aged 80 years and older, worse hearing, both continuously and categorically, was not associated with frailty status.

### Odds of being frail and pre-frail by hearing aid use

Among participants with mild or greater hearing loss (*N* = 1,724, Table 3), compared to hearing aid users (*N* = 522), nonusers had more than double the odds of being frail versus robust (OR = 2.54 [1.54–4.18]) and 51% greater odds of being pre-frail versus robust (OR = 1.51 [1.05–2.17]) in the fully adjusted model. In addition, nonusers had 68% greater odds of being frail versus pre-frail compared to users (OR = 1.68 [1.04–2.71]).

**Table 3:** Odds of being frail and pre-frail by hearing aid use status among community-dwelling older adults with hearing loss in NHATS

**Among Older Adults with Mild or Greater Hearing Loss** (*N* = 1,724)			
	**Frail** (*n* = 349) **vs. Robust** (*n* = 493)	**Pre-frail** (*n* = 882) **vs. Robust** (*n* = 493)	**Frail** (*n* = 349) **vs. Pre-frail** (*n* = 882)
**Odds Ratios (95% Confidence Intervals)**			
**Hearing aid users** (*n* = 522)	Reference	Reference	Reference
**Nonusers** (*n* = 1,202)	**2.54 (1.54, 4.18)**	**1.51 (1.05, 2.17)**	**1.68 (1.04, 2.72)**
**Among Older Adults with Moderate or Greater Hearing Loss (*N* = 864)**			
	**Frail** (*n* = 205) **vs. Robust** (*n* = 205)	**Pre-frail** (*n* = 454) **vs. Robust** (*n* = 205)	**Frail** (*n* = 205) **vs. Pre-frail** (*n* = 454)
**Odds Ratios (95% Confidence Intervals)**			
**Hearing aid users** (*n* = 408)	Reference	Reference	Reference
**Nonusers** (*n* = 456)	**2.11 (1.18, 3.77)**	1.26 (0.84, 1.90)	**1.67 (1.01, 2.78)**

Reported as relative odds ratios from a three-category multinomial model (robust, pre-frail, or frail)

Adjusted for age, sex, race/ethnicity, educational attainment, log of income, BMI, hypertension, diabetes, stroke, and BPTA

Similarly, among participants with moderate or greater hearing loss (*N* = 864, Table 3), nonusers had greater odds of being frail versus robust (OR = 2.11 [1.18–3.77] and frail versus pre-frail (OR = 1.67 [1.01–2.78]) compared to hearing aid users. The association with being pre-frail versus robust was not statistically significant (OR = 1.26 [0.84–1.90]).

### Sensitivity analyses

Participants with insufficient frailty data excluded from the primary analytic sample were similar to the frail group but were younger and had less hearing loss (Table 4). When we included them as pre-frail (Supplementary Tables S3-S4) or frail (Supplementary Tables S5-S6), findings were in the same direction; worse hearing was associated with greater odds of being frail versus robust and hearing aid users had lower odds compared to nonusers. Use of the new WHO cut points renders any effects using hearing loss categories statistically insignificant (Table S7).

**Table 4:** Characteristics of community-dwelling older adults from the National Health and Aging Trends Study (2021) stratified by frailty phenotype category (*N* = 2,510)

	**Total**	**Robust**	**Pre-frail**	**Pre-frail/Frail undecided (excluded from analytic sample)**	**Frail**	***p*-value**
Number of observations, n (%)	2,510 (100)	764 (30)	1,173 (47)	149 (6)	424 (17)	
Age, mean (SD)	81.1 (6.1)	79.1 (5.1)	81.5 (6.1)	81.5 (6.0)	83.4 (6.5)	< 0.001
Female, n (%)	1,405 (56.0)	390 (51.0)	654 (55.8)	91 (61.1)	270 (63.7)	< 0.001
Race/ethnicity, n, (%)						< 0.001
White	1,855 (73.9)	621 (81.3)	867 (73.9)	93 (62.4)	274 (64.6)	
Black	489 (19.5)	101 (13.4)	230 (19.6)	43 (28.9)	114 (26.9)	
Other^a^	166 (6.6)	41 (5.4)	76 (6.5)	13 (8.7)	36 (8.5)	
Educational attainment, n, (%)						< 0.001
Less than high school	380 (15.1)	61 (8.0)	180 (15.3)	40 (26.8)	99 (23.3)	
High school	647 (25.8)	164 (21.5)	306 (26.1)	48 (32.2)	129 (30.4)	
Some college	692 (27.6)	214 (28.0)	326 (27.8)	37 (24.8)	115 (27.1)	
College or more	791 (31.5)	325 (42.5)	361 (30.8)	24 (16.1)	81 (19.1)	
Total reported income, mean (SD)	67556.6 (149107.4)	86447.1 (97199.4)	65213.0 (189229.5)	57956.7 (151567.9)	43375.4 (79397.1)	< 0.001
Hearing aid use, n, (%)	566 (22.5)	192 (25.1)	266 (22.7)	33 (22.1)	75 (17.7)	0.034
Body Mass Index (kg/m^2^), mean (SD)	27.7 (6.1)	27.5 (4.9)	27.5 (5.8)	30.6 (10.4)	27.9 (6.7)	< 0.001
Hypertension, n (%)	1,915 (76.3)	529 (69.2)	901 (76.8)	132 (88.6)	353 (83.3)	< 0.001
Diabetes, n (%)	759 (30.2)	172 (22.5)	350 (29.8)	68 (45.6)	169 (39.9)	< 0.001
Stroke, n (%)	49 (2.0)	6 (0.8)	22 (1.9)	4 (2.7)	17 (4.0)	0.002
Hearing loss categories						< 0.001
No hearing loss (≤ 25 dB HL)	677 (27.0)	271 (35.5)	291 (24.8)	40 (26.8)	75 (17.7)	
Mild hearing loss (26–40 dB HL)	916 (36.5)	288 (37.7)	428 (36.5)	56 (37.6)	144 (34.0)	
Moderate or greater hearing loss (> 40 dB HL)	917 (36.5)	205 (26.8)	454 (38.7)	53 (35.6)	205 (48.3)	

^a^Other: Hispanic and non-Hispanic American Indian, Asian, Native Hawaiian, and Pacific Islander

## Discussion

In a nationally representative sample of older adults in the U.S., worse hearing continuously was associated with greater odds of being frail and pre-frail versus robust after adjusting for sociodemographic and health characteristics. Across hearing categories, those with moderate or greater hearing loss had 84% and 46% greater odds of being frail and pre-frail versus robust, respectively, compared to those with no hearing loss. In age-stratified analyses, the association of worse hearing with being frail versus robust remained significant among older adults aged 71–80 only. Furthermore, lack of hearing aid use was associated with greater odds of being frail and pre-frail versus robust, and frail versus pre-frail. Our findings add to our understanding of the association of hearing loss and hearing interventions with different frailty stages among older adults.

Our findings are consistent with literature on the association of hearing loss with greater odds of being frail [13]. Most studies used self-reported hearing [24–27] while few used pure-tone audiometry to investigate this association [28, 29]. A study in Spain found that moderate or greater hearing loss was associated with 85% greater odds of being frail versus non-frail, meaning having < 3 physical frailty components present (OR = 1.85 [0.98–3.49]) [29]. Longitudinal analysis of older adults from the Health, Aging, and Body Composition study found that worse hearing increases frailty risk after 10-years (Hazard Ratio [HR] = 1.11 [1.03–1.19] per 10-dB HL), with frailty defined by slow gait speed or inability to rise from a chair without using arms [28]. Despite using different frailty criteria or considering frailty as binary and overlooking the pre-frail stage, results are consistent on the association of worse hearing and being frail. Although findings were not statistically significant using the new WHO cut points, this may be because using these cut points renders hearing loss almost ubiquitous among this population of older adults, leading to a small reference group with no hearing loss. Nonetheless, the model using hearing loss as a continuous variable is significant, indicating that worse hearing is associated with increased likelihood of being frail versus robust. Importantly, key strengths of our study were the inclusion of underrepresented groups of older adults that are often excluded or not prospectively sampled in previous studies, such as the oldest old and older Black Americans, and those who may otherwise be missed due to mobility or travel restrictions, which coupled with the incorporation of sampling weights into our analysis make our findings more generalizable to the older US population.

Another major strength of this work was the use of a multinomial model with frailty status categorized into three stages as opposed to a binary variable, a novel and more granular approach allowing us to better understand the association of hearing loss with both frailty and pre-frailty. In this study, approximately half of the participants were pre-frail. Pre-frail older adults are at increased risk for outcomes including falls, mobility difficulties, hospitalization, and death, although the risk is lower compared to those who are frail [4]. They are also at risk of subsequently becoming frail [4]. However, studies on frailty transitions found that those who are pre-frail are more likely to regress than those who are frail, and therefore may be a better stage for interventions [30–32]. For example, interventions relating to nutrition, exercise, and polypharmacy may improve physical function and frailty status among pre-frail older adults [33, 34]. We found that worse hearing was associated with greater odds of being pre-frail versus robust, and it is possible that there may be a role in addressing hearing loss as part of frailty prevention interventions, although further longitudinal research is needed to establish these associations.

How hearing loss may be linked to frailty in older adults is not clear. Hearing loss impairs encoding of sound resulting in difficulty with communication and hinders spatial awareness. This may eventually lead to cognitive and physical decline via constantly high cognitive load due to effortful listening [35], greater risk for social isolation [36] and depression [37], and a poorer physical function profile including slower gait speed [10], lower levels of physical activity [9, 38], and more falls [8]. Furthermore, these manifestations may negatively impact and reinforce one another in a vicious cycle. For example, hearing loss may cause social withdrawal which in turn may contribute to physical decline and more social withdrawal. Therefore, the underlying mechanisms potentially linking hearing loss to frailty may be related to cognitive and physical decline. Interestingly, we found that the studied associations were stronger among participants ages 71–80 years, suggesting that hearing loss at a relatively younger age may have a greater impact on frailty risk. However, our findings were limited by a small sample size among the older age groups; future research is needed to better understand the role of age in the association of hearing loss and frailty.

Few studies investigated whether hearing aid use changed the studied association among older adults with hearing loss. Findings by Kamil et al. were in the direction of lower odds of frailty among hearing aid users with self-reported hearing impairment but were not statistically supported (OR = 0.82 [0.50–1.35] among men, OR = 0.44 [0.14–1.39] among women) [24]. Similarly, frailty risk among hearing aid users with measured moderate or greater hearing loss was not significantly lower (HR = 0.81 [0.54–1.21]) [28]. However, the lack of statistical significance could be related to the use of non-validated measures of frailty, sample size, or the limitation to ages 70–79 in the latter study since hearing aid use may play a more important role at older ages or after a longer duration of use. Our findings support an association with greater odds of frailty and pre-frailty among older adults with hearing loss who don’t use hearing aids. Although the estimate for pre-frail versus robust was no longer statistically significant when we limited to participants with moderate or greater hearing loss, this may be because of reduced statistical power and the comparison group being generally less healthy after excluding nonusers with mild hearing loss. Hearing aid use enhances auditory and cognitive processing which are often distorted with hearing loss, and therefore could improve cognitive [15, 39, 40] and physical function [10]. As a result, if hearing loss is truly a risk factor for frailty, hearing interventions may have the potential to modify this association. However, the cross-sectional, observational nature of this study makes it difficult to assess whether hearing aids truly have an impact on frailty, given that it may be a proxy for many things. Hearing aid use is indicative of higher socioeconomic status given the hearing healthcare disparities in the US and may therefore reflect better healthcare access and health, leading to lower risk of frailty. Furthermore, it is also possible that this association is in the opposite direction and that older adults who are frail or pre-frail are less able to obtain hearing aids. Future longitudinal studies are warranted to understand the association of hearing loss and hearing aids with frailty.

The potential role of hearing loss as a risk factor for frailty has broader implications. Currently, the United States Preventive Services Task Force (USPSTF) does not recommend screening for hearing loss in older adults as current evidence is insufficient to assess the balance of harms and benefits [41]. However, provider education on the co-occurrence of frailty and hearing loss in older adults may prompt geriatricians to become more attuned to this potential association and elicit screening for hearing loss as part of general care and potentially, if future evidence is supportive, frailty management strategies. Furthermore, if future longitudinal studies establish hearing loss as a risk factor and randomized controlled trials support hearing aids as beneficial for prevention of frailty, coverage of hearing aids could eventually be cost-effective for Medicare from a preventative perspective, beyond hearing-related health.

Our study has several strengths. First, the study sample was more inclusive of older adults in the US compared to previous studies. Second, the newly added pure-tone audiometry measures represent the most used clinical standard. Third, we present novel findings on the association of hearing loss with frailty and pre-frailty as two distinct stages. Limitations include the cross-sectional nature of this study, which prevents us from determining temporality in the association of both hearing loss and hearing aid use with frailty. Second, information on duration of hearing aid use is lacking, limiting our ability to make inferences on the role played. Third, there may be residual unmeasured confounding factors, and this may be particularly significant in the analysis comparing hearing aid users to nonusers. They are distinct groups of individuals that may significantly differ in various aspects including socioeconomic status, healthcare access, and health-seeking behaviors, that we may not have fully accounted for in our analyses. Our findings may also be influenced by survivorship bias. Lastly, excluding participants with insufficient frailty data may have biased our findings. However, in sensitivity analyses including these participants, findings were generally in the same direction.

## Conclusion

Worse hearing is associated with greater odds of being frail and pre-frail relative to robust in a nationally representative sample of older adults in the U.S. Frailty and pre-frailty are associated with mortality, and identifying and addressing modifiable risk factors is highly important. Future longitudinal research is needed to establish whether hearing loss is a risk factor for frailty.

### Supplementary Information

**Additional file 1.** Supplementary Material.

## Data Availability

The datasets generated and/or analyzed during the current study are available in the NHATS repository, www.nhats.org.
