# Supplementary material for: The association of hearing loss with frailty among community-dwelling older adults: findings from the National Health and Aging Trends Study
Source: BMC Geriatr. 2023 Nov 17;23:754. doi: 10.1186/s12877-023-04465-1 (PMC10656913; doi:10.1186/s12877-023-04465-1)
Supplement: Supplementary file 1 — Additional file 1. Supplementary Material. [file 12877_2023_4465_MOESM1_ESM.docx]

**Supplementary Material**

**Table S1. Bottom Quintile of Grip Strength by Sex and BMI in Round 11 of NHATS**

| **Sex and BMI Categories** | **Grip Strength (kg) Cut-Off for Bottom Quintile** | **Number of Participants Below the Bottom Quintile** |
| --- | --- | --- |
| Female, BMI < 18.5 kg/m^2^ | 8.3 | 11 |
| Female, BMI 18.5 to < 25 kg/m^2^ | 15 | 151 |
| Female, BMI 25 to < 30 kg/m^2^ | 15.2 | 119 |
| Female, BMI ³ 30 kg/m^2^ | 15.5 | 125 |
| Male, BMI < 18.5 kg/m^2^ | 27.5 | 4 |
| Male, BMI 18.5 to < 25 kg/m^2^ | 24 | 104 |
| Male, BMI 25 to < 30 kg/m^2^ | 26.3 | 128 |
| Male, BMI ³ 30 kg/m^2^ | 24.4 | 74 |
| Participants who were at or below the bottom quintile within each sex/BMI category were considered as having the “weakness” component of the physical frailty phenotype. | | |

**Table S2. Bottom Quintile of Walking Speed by Sex and Height in Round 11 of NHATS**

| **Sex and Height Categories** | **Walking Speed (m/s) Cut-Off for Bottom Quintile** | **Number of Participants Below the Bottom Quintile** |
| --- | --- | --- |
| Female, ≤ 159 cm | 0.43 | 196 |
| Female, >159 cm | 0.60 | 421 |
| Male, ≤ 159 cm | 0.50 | 132 |
| Male, >159 cm | 0.60 | 244 |
| Participants who were at or below the bottom quintile within each sex/height category were considered as having the “slow walking speed” component of the physical frailty phenotype. | | |

**Table S3. Odds of being frail and pre-frail by hearing status among older adults in NHATS (N=2,510, excluded participants added to the pre-frail category)**

|  | **Frail vs. Robust** | **Pre-frail vs. Robust** | **Frail vs. Pre-frail** |
| --- | --- | --- | --- |
|  | **Odds Ratios (95% Confidence Intervals)** | | |
| **BPTA^a^, per 10 dB HL^b^** | 1.20 (1.05, 1.38) | 1.09 (0.99, 1.20) | 1.11 (0.97, 1.26) |
| **Hearing loss categories** |  |  |  |
| No hearing loss | Reference | Reference | Reference |
| Mild hearing loss | 1.32 (0.85, 2.07) | 1.25 (0.92, 1.70) | 1.06 (0.71, 1.58) |
| Moderate or greater hearing loss | 1.84 (1.09, 3.10) | 1.40 (0.97, 1.99) | 1.32 (0.81, 2.17) |
| Adjusted for age, sex, race/ethnicity, educational attainment, log of income, BMI, hypertension, diabetes, and stroke.  Reported as relative odds ratios from three-category multinomial model (robust, pre-frail, or frail)  Abbreviations: ^a^BPTA, better-hearing ear pure-tone average; ^b^dB HL, decibels hearing level | | | |

**Table S4. Odds of being frail and pre-frail by hearing aid status among older adults in NHATS (N=1,833, excluded participants added to the pre-frail category)**

|  | **Frail vs. Robust** | **Pre-frail vs. Robust** | **Frail vs. Pre-frail** |
| --- | --- | --- | --- |
|  | **Odds Ratios (95% Confidence Intervals)** | | |
| **Nonusers** | Reference | Reference | Reference |
| **Hearing aid users** | 2.52 (1.54, 4.15) | 1.49 (1.05, 2.12) | 1.69 (1.06, 2.70) |
| Adjusted for age, sex, race/ethnicity, educational attainment, log of income, BMI, hypertension, diabetes, stroke, and BPTA.  Reported as relative odds ratios from three-category multinomial model (robust, pre-frail, or frail). | | | |

**Table S5. Odds of being frail and pre-frail by hearing status among older adults in NHATS (N=2,510, excluded participants added to the frail category)**

|  | **Frail vs. Robust** | **Pre-frail vs. Robust** | **Frail vs. Pre-frail** |
| --- | --- | --- | --- |
|  | **Odds Ratios (95% Confidence Intervals)** | | |
| **BPTA^a^, per 10 dB HL^b^** | 1.15 (1.02, 1.29) | 1.09 (0.99, 1.20) | 1.05 (0.94, 1.16) |
| **Hearing loss categories** |  |  |  |
| No hearing loss | Reference | Reference | Reference |
| Mild hearing loss | 1.18 (0.77, 1.80) | 1.28 (0.94, 1.75) | 0.92 (0.63, 1.33) |
| Moderate or greater hearing loss | 1.50 (0.96, 2.34) | 1.44 (1.00, 2.09) | 1.04 (0.69, 1.56) |
| Adjusted for age, sex, race/ethnicity, educational attainment, log of income, BMI, hypertension, diabetes, and stroke.  Reported as relative odds ratios from three-category multinomial model (robust, pre-frail, or frail)  Abbreviations: ^a^BPTA, better-hearing ear pure-tone average; ^b^dB HL, decibels hearing level | | | |

**Table S6. Odds of being frail and pre-frail by hearing aid status among older adults in NHATS (N=1,833, excluded participants added to the frail category)**

|  | **Frail vs. Robust** | **Pre-frail vs. Robust** | **Frail vs. Pre-frail** |
| --- | --- | --- | --- |
|  | **Odds Ratios (95% Confidence Intervals)** | | |
| **Nonusers** | Reference | Reference | Reference |
| **Hearing aid users** | 2.09 (1.39, 3.13) | 1.51 (1.05, 2.17) | 1.39 (0.95, 2.03) |
| Adjusted for age, sex, race/ethnicity, educational attainment, log of income, BMI, hypertension, diabetes, stroke, and BPTA.  Reported as relative odds ratios from three-category multinomial model (robust, pre-frail, or frail). | | | |

**Table S7. Odds of Being Frail and Pre-frail by Hearing Status Among Community-Dwelling Older Adults from NHATS Using 2021 WHO Categories**

|  | **Frail** (n=424)  **vs. Robust** (n=764) | **Pre-frail** (n=1,173)  **vs. Robust** (n=764) | **Frail** (n=424)  **vs. Pre-frail** (n=1,173) |
| --- | --- | --- | --- |
|  | **Odds Ratios [95% Confidence Intervals]** | | |
| **Hearing loss categories (WHO, 2021)** |  |  |  |
| No hearing loss (<20 dB HL^b^, **n=295**) | Reference | Reference | Reference |
| Mild hearing loss (20-34.9 dB HL^b^, **n=868**) | 1.23 [0.61-2.51] | 1.03 [0.72-1.48] | 1.20 [0.59-2.42] |
| Moderate hearing loss (35-49.9 dB HL^b^, **n=754**) | 1.58 [0.74-3.40] | 1.45 [0.89-2.37] | 1.09 [0.56-2.13] |
| Moderately severe hearing loss (50-64.9 dB HL^b^, **n=342**) | 1.91 [0.81-4.51] | 1.04 [0.58-1.85] | 1.85 [0.79-4.34] |
| Severe or greater hearing loss (>64.9 dB HL^b^, **n=102**) | 2.45 [0.86-6.97] | 1.47 [0.72-2.97] | 1.67 [0.62-4.49] |
| Reported as relative odds ratios from a three-category multinomial model (robust, pre-frail, or frail). Adjusted for age, sex, race/ethnicity, educational attainment, log of income, BMI, hypertension, diabetes, and stroke.  Abbreviations: ^a^BPTA, better-hearing ear pure-tone average; ^b^dB HL, decibels hearing level | | | |
